# Entropy-Based Image Fusion with Joint Sparse Representation and Rolling Guidance Filter

**DOI:** 10.3390/e22010118

**Published:** 2020-01-18

**Authors:** Yudan Liu, Xiaomin Yang, Rongzhu Zhang, Marcelo Keese Albertini, Turgay Celik, Gwanggil Jeon

**Affiliations:** 1 College of Electronics and Information Engineering, Sichuan University, Chengdu 610064, China; shanke27@126.com (Y.L.); zhang_rz@scu.edu.cn (R.Z.); 2 Department of Computer Science, Federal University of Uberlandia, Uberlandia, MG 38408-100, Brazil; albertini@ufu.br; 3 School of Computer Science and Applied Mathematics, University of the Witwatersrand, Johannesburg 2000, South Africa; celikturgay@gmail.com; 4 School of Electronic Engineering, Xidian University, Xi’an 710071, China; 5 Department of Embedded Systems Engineering, Incheon National University, Incheon 22012, Korea

**Keywords:** image entropy, joint bilateral filter, image fusion, rolling guidance filter, joint sparse representation, multi-scale decomposition

## Abstract

Image fusion is a very practical technology that can be applied in many fields, such as medicine, remote sensing and surveillance. An image fusion method using multi-scale decomposition and joint sparse representation is introduced in this paper. First, joint sparse representation is applied to decompose two source images into a common image and two innovation images. Second, two initial weight maps are generated by filtering the two source images separately. Final weight maps are obtained by joint bilateral filtering according to the initial weight maps. Then, the multi-scale decomposition of the innovation images is performed through the rolling guide filter. Finally, the final weight maps are used to generate the fused innovation image. The fused innovation image and the common image are combined to generate the ultimate fused image. The experimental results show that our method’s average metrics are: mutual information (MI)—5.3377, feature mutual information (FMI)—0.5600, normalized weighted edge preservation value ($Q^{AB/F}$)—0.6978 and nonlinear correlation information entropy (NCIE)—0.8226. Our method can achieve better performance compared to the state-of-the-art methods in visual perception and objective quantification.

## 1. Introduction

Image fusion combines multiple source images of the same scene together to make the fusion image more suitable for human visual perception or computer processing [1]. The source images are obtained from different sensors or imaging conditions. Each source image contains redundant and complementary information about the scene. The purpose of image fusion is to integrate the redundant and complementary information to make the fused image contain more relevant information specific to an application or task. Image fusion technology has good application prospects. It has been applied in many fields. In the medical field, we can reconstruct fused images by fusing multiple medical images from different sensors. The fused images are able to provide complementary information for medical analysis, enabling doctors to diagnose more quickly and accurately [2]. Moreover, surveillance is a typical application of image fusion [3]. Using an infrared–visible image fusion, a surveillance system can work effectively all day.

Image fusion methods based on transform domain can be divided into two types: multi-scale decomposition and sparse representation [4]. Multi-scale decomposition (MSD) is a very classical approach to performing image fusion. The basic process of MSD-based methods contains three steps. First, source images are converted into a specific transform domain, which typically decomposes the source images into multi-scale representations. Then, different fusion strategies are adopted for each scale level to acquire the multi-scale representation of the fused image in the transform domain. Finally, the corresponding inverse transformation is utilized to reconstruct the fused image. Traditional MSD methods can be divided into two categories. One includes the pyramid-based methods such as Laplacian pyramid (LP) [5] and gradient pyramid (GP) [6]. The other contains the wavelet transform-based methods, such as discrete wavelet transform (DWT) [7] and dual-tree complex wavelet transform (DTCWT) [8]. In addition, there are some new MSD methods, such as non-subsampled contourlet transform (NSCT) [9], shift-invariant shearlet transform (SIST) [10], non-subsampled shearlet transform (NSST) [11] and complex shearlet transform (CST) [12]. In recent years, the edge- preserving filter based-MSD has been a hot research direction. Edge-preserving smoothing filters, such as the guided filter (GF) [13], joint bilateral filter (JBF) [14] and rolling guidance filter (RGF) [15], can avoid ringing artifacts, since they do not blur strong edges in the decomposition process. Li et al. put forward a method of image fusion using GF [16]. Chen et al. proposed an infrared-visible image fusion method combining RGF and multi-directional decomposition [17]. Jian et al. combined RGF and JBF together for image fusion [18]. Although these methods achieve quite good performance on many types of images, they still have the following disadvantages: (1) the redundant information between source images leads to low information entropy of fused images; (2) contrast of fused images is likely to decrease.

Sparse representation (SR) is another classic image processing method. SR conforms to the physiological characteristics of human vision [19]. Besides, it has robustness to additive noise [20]. SR has been successfully applied in many image processing fields, such as image classification [21], image denoising [22], image communication [23] and image super-resolution [24]. Yang and Li [25] were the first ones to utilize SR to deal with image fusion problem. The general flow of SR-based methods contains three steps. First, an appropriate over-complete sparse dictionary is constructed through a mathematical model or example learning. Second, the sparse representations of the source images on the given sparse dictionary are obtained. The sparse representations are usually sets of sparse coefficient vectors. Finally, the corresponding vectors are fused to get the coefficient vector set of the fused image. The fused image is reconstructed with the same dictionary. Recently, a new SR-based method called joint sparse representation (JSR) was applied to image fusion successfully. The idea of JSR is to use sparse representation to divide two source images into a common part and two innovation parts. The common part contains the redundant information shared by the two source images, while the two innovation parts represent the complementary information of each source image. After that, different fusion rules are used to fuse the two kinds of part separately. JSR preserves the advantages of the SR method while eliminating the correlation between source images so that the fused image is less affected by the redundant information. Yu et al. proposed a JSR-based approach to carry out image denoising and fusion simultaneously [26]. Ma et al. combined JSR and optimum theory to address the multi-focus image fusion problem [27]. However, including JSR, all SR-based methods generally have the following disadvantages: (1) an over-complete dictionary may result in visual artifacts in the reconstructed image; (2) simple fusion strategy for sparse coefficient vectors leads to spatial inconsistency.

In order to make full use of the advantages and overcome the shortcomings of the above methods, we propose a new image fusion method combining JSR and MSD. Specifically, first, JSR is used to decompose two source images into a common image and two innovation images. Then, the innovation images are sent to RGF-based MSD fusion framework to obtain the fused innovation image. Finally, the fused innovation image and the common image are combined to obtain the fused image.

The main contributions of our work are as follows:

1. To improve the low information entropy caused by the redundant information between source images, only innovation images are performed edge-preserving MSD through RGF.
2. To suppress the artifacts that may be brought by JSR, weight maps are used to balance the contribution of innovation images.
3. To make fused images have high contrast, innovation images are used to guide the optimization of the weight maps.
4. To ensure the spatial consistency of fused images, the fusion of innovation images is performed according to optimized weight maps.

## 2. Related Work

### 2.1. Joint Sparse Representation

JSR is a novel method with which to perform image fusion. As with SR, JSR has two key issues: (1) sparse dictionary construction; (2) joint sparse coding to obtain coefficients. For JSR, constructing an over-complete sparse dictionary is exactly the same as in SR. In this study, we used K-SVD [28] to train the dictionary.

The biggest difference between JSR and SR is the method of sparse coding. In image processing, the objects for sparse coding are overlapping small image patches of source images. These patches are extracted by the sliding-window technique. The small patches of all the source images at the same position constitute a patch set. SR encodes each patch separately, while JSR simultaneously encodes all patches in the same set. JSR supposes that every patch consists of a common component and an innovation component. The common component is shared by all patches in the same set, while each patch has its own innovation component. There are two strategies for selecting the dictionaries for JSR. One strategy is to use one fixed dictionary for all the components for sparse encoding and reconstruction [26]. This strategy has low training cost, is easy to operate, and is suitable for the sparse representation of multiple types of images. The other strategy is to use a fixed dictionary for common components and an adaptive dictionary for innovation components for sparse coding and reconstruction [27]. This strategy may yield better results, but comes with additional computational costs. Our method uses the first strategy, which is to use a fixed dictionary for all components. Since there are always two source images for fusion, suppose each patch set has *W* patches. Given a flatten patch $v_{i}\in R^{n}\left(1\leq i\leq 2\right)$ extracted from a source image and an over-complete dictionary $D\in R^{n\times m}\left(n<m\right)$, where *n* represents the length of the vector and *m* represents the number of atoms in the dictionary, respectively. The goal of JSR is to estimate a common sparse vector $x^{C}\in R^{m}$ and innovation sparse vectors $x_{i}^{I}\in R^{m}\left(1\leq i\leq 2\right)$ with only a few nonzero entries, such that

(1) $$v_{i}\approx Dx^{C}+Dx_{i}^{I},$$

where $Dx^{C}$ and $Dx_{i}^{I}$ represent the common component and the innovation component of $v_{i}$, respectively.

When performing sparse coding, JSR needs to first concatenate the source image patches and the dictionary separately. The encoded sparse coefficient vectors are also concatenated. Let

$$V=\left[\begin{array}{c} v_{1}\\ v_{2} \end{array}\right],\;\tilde{D}=\left[\begin{array}{ccc} D & D & 0\\ D & 0 & D \end{array}\right],\;X=\left[\begin{array}{c} x^{C}\\ x_{1}^{I}\\ x_{2}^{I} \end{array}\right],$$

where *V* denotes the concatenated source patch, $\tilde{D}$ denotes the concatenated dictionary and *X* denotes the concatenated sparse coefficient vector. The joint version of Equation (1) can be defined as follows:

(2) $$V\approx \tilde{D}X.$$

The goal of JSR is to find an approximate optimal solution of *X*. This problem can be formulated as

(3) $$\arg\min_{X}{∥X∥}_{0}\;s.t.\;{∥\tilde{D}X-V∥}_{2}\leq \varepsilon ,$$

where ε is a sparse reconstruction error.

Our proposed method uses JSR as an image decomposition method to reduce correlations between source images and increase the information entropy of a fused image. First, the two source images are divided into small overlapping image patches through the sliding window technique. The dimensions of the patches depends on the specific dictionary. Assuming the dictionary $D\in R^{n\times m}$, then the size of each patch should be $\sqrt{n}\times \sqrt{n}$. Every patch is rearranged to a column vector. Second, two patches located at the same position compose a patch set. JSR is performed independently on each patch set. For every patch set, a common sparse vector belonging to the entire set and two innovative sparse vectors belonging to each patch in the set can be obtained. Then, a common patch and two innovation patches can be reconstructed by the corresponding sparse vector for each set. Finally, all the common patches and the corresponding innovation patches are averaged in the same order they were selected during the sliding window step. A common image and two innovation images are generated. The correlation between the two innovation images is lower than that between the two source images. We define the process of obtaining a common image and innovation images from source images as JSR decomposition.

### 2.2. Rolling Guidance Filter

The purpose of multi-scale decomposition is to obtain images of different blur levels to make full use of the information contained in the source images. Recently, edge-preserving filter-based MSD methods have become the mainstream of research. These methods are able to preserve high-contrast edges and obvious structures while blurring the image. The state-of-the-art edge-preserving MSD method was proposed by by Jian et al. [18]. It uses a rolling guidance filter (RGF).

A RGF can be seen as an extension of joint bilateral filter (JBF). JBF is first proposed for image denoising by Petschnigg et al. [14]. JBF accepts an input image and a guidance image as input. The content of the output image is similar to the input image, while the structures and edges of it are similar to the guidance image. First, the Gaussian kernel $g_{\delta }$ is given as:

(4) $$g_{\delta }\left(p,q\right)=\exp\left(-\frac{{∥p-q∥}^{2}}{2\delta^{2}}\right),$$

where *p* and *q* index pixel coordinates in the image, and δ is the standard deviation. With Equation (4), given a guidance image *G* and an input image $I_{in}$, the definition of JBF is as follows:

(5) $$I_{out}\left(p\right)=\frac{1}{U}\sum_{q\in N\left(p\right)}g_{\delta_{s}}\left(p,q\right)g_{\delta_{r}}\left(G\left(p\right),G\left(q\right)\right)I_{in}\left(q\right),$$

where $I_{out}$ is the output image, $U=\sum_{q\in N\left(p\right)}g_{\delta_{s}}\left(p,q\right)g_{\delta_{r}}\left(G\left(p\right),G\left(q\right)\right)$ is the normalization factor, $N\left(p\right)$ is the set of neighboring pixels of *p*, $\delta_{s}$ is the spatial standard deviation and $\delta_{r}$ is the range standard deviation. The function $g_{\delta_{s}}$ sets the weight in the spatial domain based on the distance between the pixels, while the function $g_{\delta_{r}}$ sets the weight on the range based on intensity differences. $\delta_{s}$ and $\delta_{r}$ control the spatial and range weights, respectively. For the sake of simplicity, we abbreviate Equation (5) as follows:

(6) $$I_{out}=JBF\left(I_{in},G,\delta_{s},\delta_{r}\right),$$

where $JBF\left(\cdot \right)$ denotes the JBF process.

**Figure 1:**
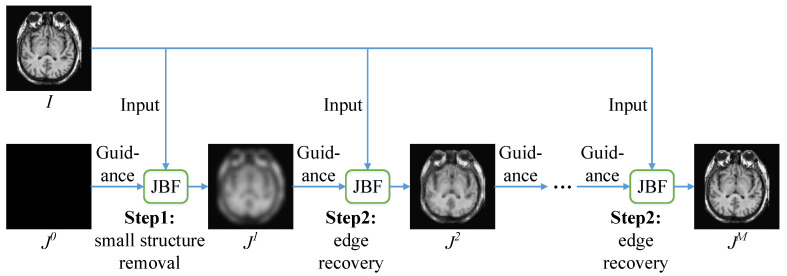
Rolling guidance filtering.

RGF was proposed by Zhang et al. [15]. The biggest feature of RGF is the ability to remove small structures while preserving the main content of the image. RGF is composed of two main steps: small structure removal and edge recovery. As shown in Figure 1, RGF is an iterative process. Suppose $J^{t}$ is the result in the *t*-th iteration and *M* is the number of total iterations, the iterative process of RGF is as follows:

(7) $$J^{t}=JBF\left(I_{in},J^{t-1},\sigma_{s},\sigma_{r}\right),\;t=1,2,\cdots ,M,$$

where $\sigma_{s}$ and $\sigma_{r}$ denote the standard deviations of RGF in order to distinguish them from $\delta_{s}$ and $\delta_{r}$ of JBF.

According to Equation (7), the iteration process of RGF is given as Algorithm 1.
**Algorithm 1: The iteration process of RGF.****Input:** Input image $I_{in}$; spatial standard deviation $\sigma_{s}$; range standard deviation $\sigma_{r}$; iteration number *M*. 1: Set $J^{0}$ as a constant image, i.e., $\forall p,J^{0}\left(p\right)=C$, where *C* is a constant value. 2: **for**
t=1:1:M
**do** 3:  $J^{t}=JBF\left(I_{in},J^{t-1},\sigma_{s},\sigma_{r}\right)$. 4: **end for****Output:** Output image $I_{out}=J^{M}$.

Since $J^{0}$ is set as a constant image, the first iteration is equivalent to blurring $I_{in}$ with a Gaussian filter with the standard deviation of $\sigma_{s}$ to remove small structures. The remaining iterations are equivalent to continuous filtering of $I_{in}$ by the joint bilateral filter. Specifically, in each iteration, JBF takes $I_{in}$ as input and $J^{t-1}$ as guidance, and $\sigma_{s}$ and $\sigma_{r}$ as standard deviations. Edges are recovered gradually during this process. After all the iterations are completed, $J^{M}$ is the output $I_{out}$.

For simplicity, we denote the RGF filtering operation as follows:

(8) $$I_{out}=RGF\left(I_{in},\sigma_{s},\sigma_{r},M\right),$$

where $RGF\left(\cdot \right)$ denotes the RGF function.

## 3. Proposed Method

**Figure 2:**
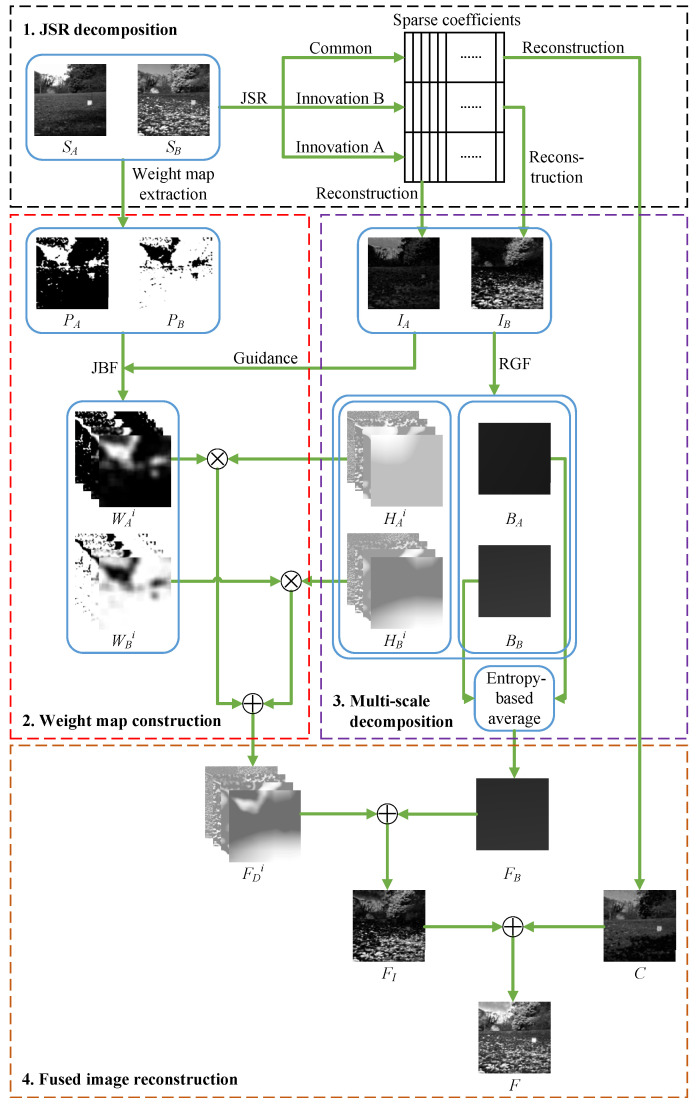
The schematic diagram of our proposed fusion method.

Our proposed method is shown in Figure 2, which contains four stages: (1) JSR decomposition; (2) weight map construction; (3) multi-scale decomposition; (4) fused image reconstruction.

### 3.1. JSR Decomposition

**Figure 3:**
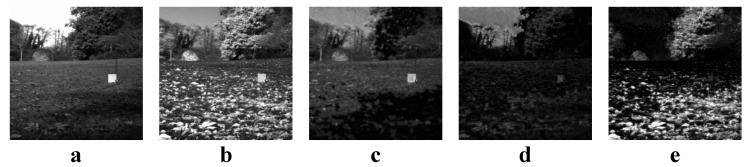
An example of JSR decomposition. (**a**,**b**) Source images; (**c**) The common image; (**d**) The innovation image of (**a**); (**e**) The innovation image of (**b**).

We first use JSR decomposition to decompose two source images $S_{A}$ and $S_{B}$ into a common image *C* and two innovation images $I_{A}$ and $I_{B}$. An example of JSR decomposition is given in Figure 3.

### 3.2. Weight Map Construction

The weight maps are references for fusing detail layers. In order to make the information accurate and complete, weight maps are generated from the source images. First, $S_{A}$ and $S_{B}$ are processed with Kirsch operator to obtain the saliency maps $R_{A}$ and $R_{B}$. Next, the initial weight maps $P_{A}$ and $P_{B}$ are obtained through a pixel-by-pixel comparison of the saliency maps $R_{A}$ and $R_{B}$ defined as follows:

(9) $$\begin{array}{c} P_{A}\left(q\right)=\left\{\begin{array}{cc} 0, & if\;R_{A}\left(q\right)\leq R_{B}\left(q\right)\\ 1, & otherwise \end{array}\right.,\\ P_{B}\left(q\right)=1-P_{A}\left(q\right), \end{array}$$

where *q* denotes pixel coordinates. The order in which the two source images are considered does not affect the initial weight maps. At each pixel position *q*, the initial weight of the one with the larger saliency value is set to 1 and the other is set to 0. If $R_{A}\left(q\right)=R_{B}\left(q\right)$, then either of the two initial weights can be set to 1 and the other is set to 0, which does not affect the result. However, it is almost impossible for two saliency values to be exactly equal.

Finally, JBF is used to filter $P_{A}$ and $P_{B}$, and the final weight maps are obtained. This step is as follows:

(10) $$\begin{array}{c} W_{A}^{i}=JBF\left(P_{A},I_{A},\delta_{s}^{i},\delta_{r}^{i}\right),\;i=1,2,\cdots ,K-1,\\ W_{B}^{i}=JBF\left(P_{B},I_{B},\delta_{s}^{i},\delta_{r}^{i}\right),\;i=1,2,\cdots ,K-1. \end{array}$$

By adjusting $\delta_{d}^{i}$ and $\delta_{r}^{i}$, weight maps for detail layers are optimized. The innovation images $I_{A}$ and $I_{B}$ are used as guidance images to enhance the difference between weight maps. This results in higher contrast in the fused image. Besides, this step makes the weight values same for the pixels with similar brightness and next to each other so that the problems caused by spatial consistency can be avoided [16].

### 3.3. Multi-Scale Decomposition

RGF is the key to performing this stage. When *M* and $\sigma_{r}$ are set to constants, changes of $\sigma_{s}$ can achieve different blur levels of rolling guidance filtering. As $\sigma_{s}$ increases, the output of RGF becomes more blurred, which means it contains more low-frequency components. Therefore, the output of using the largest $\sigma_{s}$ for RGF should be regarded as the base layer. Outputs using various smaller $\sigma_{s}$ are sequentially differentiated and the difference values are regarded as the detail layers.

Now we take $I_{A}$ as an example to give the concrete multi-scale decomposition method. First, $I_{A}$ is normalized to range $\left[0,1\right]$ and blurred into K−1 levels through RGF; this process is described by Equation (11).

(11) $$O_{A}^{i}=RGF\left(I_{A},\sigma_{s}^{i},\sigma_{r},M\right),\;i=1,2,\cdots ,K-1,$$

where $O_{A}^{i}$ denotes the outputs of RGF using different $\sigma_{s}^{i}$. Let $\sigma_{s}^{i+1}>\sigma_{s}^{i}$; then, the smooth level is $O_{A}^{i+1}>O_{A}^{i}$. Furthermore, in order to give a unified equation form, let $O_{A}^{0}=I_{A}$. Finally, base layers $B_{A}$ and K−1 detail layers $H_{A}^{i}$ $\left(i=1,2,\cdots ,K-1\right)$ can be obtained by Equation (12)

(12) $$\begin{array}{c} B_{A}=O_{A}^{K-1},\\ H_{A}^{i}=O_{A}^{i-1}-O_{A}^{i},\;i=1,2,\cdots ,K-1. \end{array}$$

The same operation is performed to $I_{B}$ to get $B_{B}$ and $H_{B}^{i}$$\left(i=1,2,\cdots ,K-1\right)$.

### 3.4. Fused Image Reconstruction

There are four steps to reconstruct the final fused image: (1) reconstruct the fused base layer $F_{B}$; (2) reconstruct the fused detail layers $F_{H}^{i}$; (3) reconstruct the fused innovation image $F_{I}$ by combining $F_{B}$ and $F_{H}^{i}$; (4) reconstruct the final fused image *F* by combining $F_{I}$ and the common image *C*.

First, the fused base layer $F_{B}$ is reconstructed by entropy-based average. The base layer contains the low-frequency information of the image, which is equivalent to the average value of the image. Fusing base layers by the traditional simple averaging method may cause the fused image have low contrast and information entropy. Since the global variance of a base layer represents its overall contrast and amount of information, we regard global variance of a base layer as its entropy. Then, an entropy-based average method is used to fuse the base layers. Specifically, the entropies $E_{A}$ and $E_{B}$ of the two base layers $B_{A}$ and $B_{B}$ are calculated by:

(13) $$\begin{array}{c} E_{A}=var\left(B_{A}\right),\\ E_{B}=var\left(B_{B}\right), \end{array}$$

where $var\left(\cdot \right)$ denotes the global variance function. Second, the fused base layer $F_{B}$ is reconstructed by weighted average using $E_{A}$ and $E_{B}$ as weights:

(14) $$F_{B}=\frac{1}{E_{A}+E_{B}}\left(E_{A}B_{A}+E_{B}B_{B}\right).$$

Second, the fused detail layers $F_{H}^{i}$ are reconstructed. The detail layers are simply multiplied by the corresponding weight maps and summed up to achieve the fusion. This process is described as follows:

(15) $$F_{H}^{i}=H_{A}^{i}W_{A}^{i}+H_{B}^{i}W_{B}^{i},\;i=1,2,\cdots ,K-1.$$

Then, with the fused detail layers $F_{H}^{i}$ and base layer $F_{B}$, the fused innovation image $F_{I}$ can be obtained as follows:

(16) $$F_{I}=F_{B}+\sum_{i=1}^{K-1}F_{H}^{i}.$$

Finally, the fused innovation image $F_{I}$ and the common image *C* are merged together to obtain the ultimate fused image *F*:

(17) $$F=F_{I}+C.$$

### 3.5. Workflow of Our Proposed Method

The workflow of our proposed method can be summarized as follows. Consider two source images $S_{A}$ and $S_{B}$, dictionary *D*, the decomposition level *K* and the parameters of JBF and RGF. First, JSR is used to decompose the two source images into one common image *C* and two innovation images $I_{A}$ and $I_{B}$. Second, the Kirsch operator is used to extract saliency maps $P_{A}$ and $P_{B}$ from source images. Regarding the innovation images as guidance, JBF is applied to the saliency maps to obtain weight maps $W_{A}^{i}$ and $W_{B}^{i}$ $\left(i=1,2,\cdots ,K-1\right)$. Then, K-level multi-scale decomposition of the innovation images is performed by RGF to obtain the detail layers $H_{A}^{i}$ and $H_{B}^{i}$ $\left(i=1,2,\cdots ,K-1\right)$ and the base layers $B_{A}$ and $B_{B}$. Finally, the detail layers are fused according to the corresponding weight maps. The base layers are fused by an entropy-based fusion rule. By summing the fused detail layers $F_{H}^{i}$ $\left(i=1,2,\cdots ,K-1\right)$ and the fused base layer $F_{B}$, the fused innovation image $F_{I}$ is obtained. The last step is to add $F_{I}$ to the common image *C*, and the fused image is obtained.

When doing addition and multiplication, the problem of dynamic range of images needs to be addressed. In our workflow, multiplication and addition occur during the fusion of the detail and base layers. For the fusion of detail layers, two detail layers on the same decomposition level are respectively multiplied with their corresponding weight maps, and the products are added to obtain the fused detail layer. The two corresponding weight maps are complementary, that is, the sum of two weights at the same pixel position is very close to 1. Therefore, the value range of the fused detail layers hardly changes. For the fusion of base layers, since the two weights are also complementary, the value range of the fused base layer does not change either. By adding all the fused detail layers and the fused base layer together, the fused image can be reconstructed. This final addition may cause a small number of pixels to be out of the reasonable range of $\left[0,255\right]$. For these pixels, the values less than 0 are set to 0 and the values greater than 255 are set to 255. Finally, the value of each pixel is rounded into an integer.

The pseudo code of our proposed method is shown as Algorithm 2.
**Algorithm 2: Pseudo code of our proposed method.****Input:** Source images $S_{A},S_{B}$; Dictionary *D*; Decomposition level *K*; JBF parameters $\delta_{s}^{i},\delta_{r}^{i}$; RGF parameters $\sigma_{s}^{i},\sigma_{r},M$.  1: Use *D* to perform JSR decomposition on $S_{A},S_{B}$ to get $C,I_{A},I_{B}$.  2: Process $S_{A},S_{B}$ with Kirsch operator to get $R_{A},R_{B}$.  3: **for**
*q* in pixel coordinate range of $R_{A}$
**do**  4:  **if**
$R_{A}\left(q\right)\leq R_{B}\left(q\right)$
**then**  5:   $P_{A}\left(q\right)=0,P_{B}\left(q\right)=1$.  6:  **else**  7:   $P_{A}\left(q\right)=1,P_{B}\left(q\right)=0$.  8:  **end if**  9: **end for** 10: **for**
i=1:1:K−1
**do** 11:  $W_{A}^{i}=JBF\left(P_{A},I_{A},\delta_{s}^{i},\delta_{r}^{i}\right)$, $W_{B}^{i}=JBF\left(P_{B},I_{B},\delta_{s}^{i},\delta_{r}^{i}\right)$. 12:  $O_{A}^{i}=RGF\left(I_{A},\sigma_{s}^{i},\sigma_{r},M\right)$, $O_{B}^{i}=RGF\left(I_{B},\sigma_{s}^{i},\sigma_{r},M\right)$. 13: **end for** 14: $B_{A}=O_{A}^{K-1}$, $B_{B}=O_{B}^{K-1}$. 15: $O_{A}^{0}=I_{A}$, $O_{B}^{0}=I_{B}$. 16: **for**
i=1:1:K−1
**do** 17:  $H_{A}^{i}=O_{A}^{i-1}-O_{A}^{i}$, $H_{B}^{i}=O_{B}^{i-1}-O_{B}^{i}$. 18: **end for** 19: $E_{A}=var\left(B_{A}\right)$, $E_{B}=var\left(B_{B}\right)$. 20: $F_{B}=\frac{1}{E_{A}+E_{B}}\left(E_{A}B_{A}+E_{B}B_{B}\right)$. 21: $F_{H}=\sum_{i=1}^{K-1}H_{A}^{i}W_{A}^{i}+H_{B}^{i}W_{B}^{i}$. 22: $F_{I}=F_{B}+F_{H}$. 23: $F=F_{I}+C$. **Output:** Fused image *F*.

## 4. Experimental Results and Analysis

### 4.1. Experimental Settings and Objective Evaluations

**Figure 4:**
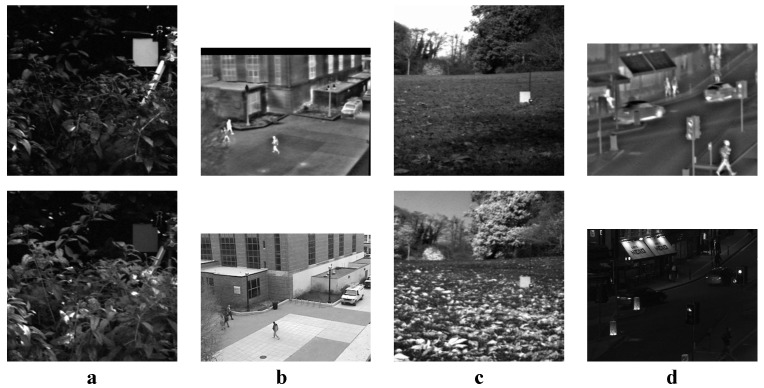
Infrared-visible image sets. (**a**–**d**) Four sets of infrared–visible source images.

**Figure 5:**
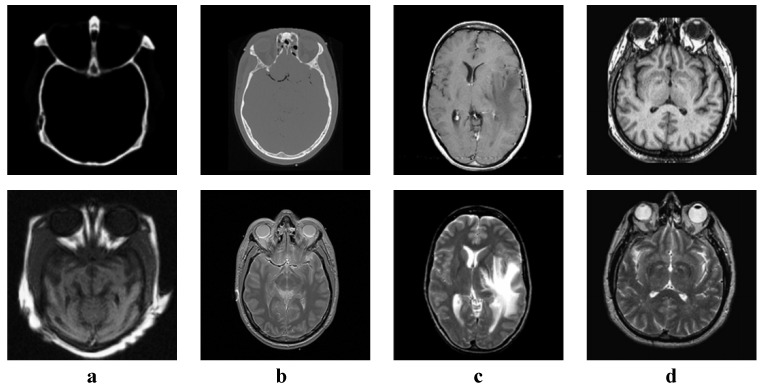
Medical image sets. (**a**–**d**) Four sets of medical source images.

**Figure 6:**
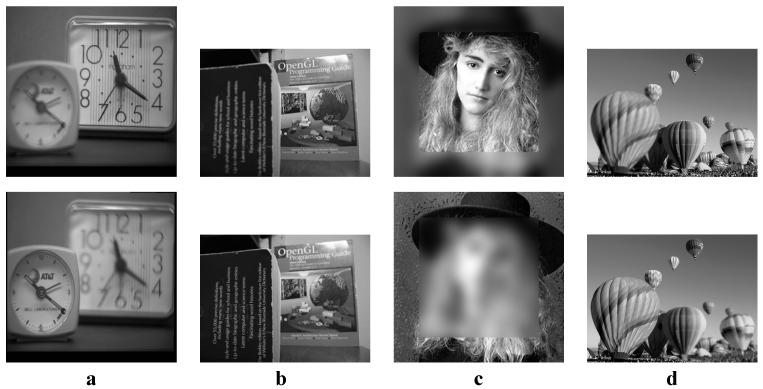
Multi-focus image sets. (**a**–**d**) Four sets of multi-focus source images.

**Figure 7:**
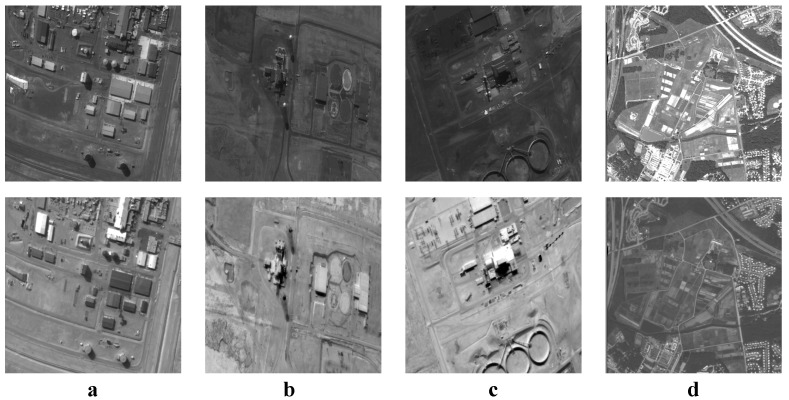
Remote sensing image sets. (**a**–**d**) Four sets of remote sensing source images.

We tested all the methods using four categories of images, with four sets of source images in each category. Specifically, there are infrared-visible images shown in Figure 4, medical images shown in Figure 5, multi-focus images shown in Figure 6 and remote sensing images shown in Figure 7. The images we used for our experiments are downloadable from the following website: https://sites.google.com/view/durgaprasadbavirisetti/datasets.

The default parameters in our method are set according to [18]. Specifically, for the parameters of JBF, we set $\delta_{s}^{i}=\left\{1,3,10,30\right\}\left(i=1,2,3,4\right)$ and $\delta_{r}^{i}=\left\{10,30,100,300\right\}\left(i=1,2,3,4\right)$ as defaults. For the RGF part, we set $\sigma_{s}^{i}=\left\{3,18,108,648\right\}\left(i=1,2,3,4\right)$, $\sigma_{r}=0.2$ and M=5 as a default. The other parameters are discussed in the following experiments. All the experiment’s programs were generated in Matlab R2019a (MathWorks, Natick, MA, USA) on an Intel(R) Core(TM)i5-6400CPU (Intel, Santa Clara, CA, USA) @ 2.70 GHz with 8.00 GB RAM.

The objective evaluation has a certain reference value for judging the quality of image fusion. In our experiments, four metrics were used to measure the information entropy and visual quality of the results of different methods:

1. Mutual information (MI) [29] based on Shannon entropy and relative entropy. It measures the correlation between the source image and the fused image to indicate how much information is retained.
2. Feature mutual information (FMI) [30] indicates the entropy of features in fused image. It measures the amount of information in image features carried from the source images to the fused image. Besides, it is a non-reference image fusion metric.
3. The normalized weighted edge preservation value ($Q^{AB/F}$) [31] measures the visual information quality of the fusion, and more edge information can lead to higher values for this metric.
4. Nonlinear correlation information entropy (NCIE) [32] is based on nonlinear joint entropy. It measures the general correlation between the source images and the fused image.

Higher values of the above four metrics demonstrate a better fusion effect. The codes of the metrics are provided by Qu et al. [33].

### 4.2. Discussions about Parameters

#### 4.2.1. Size of JSR Dictionary

This experiment tested the effect of different JSR dictionary sizes on the fusion performance. Suppose the dictionary $D\in R^{n\times m}\left(n<m\right)$; then *n* affects the size of image patches while *m* affects the completeness of the dictionary. A larger *n* indicates a larger size of image patches, which leads to an enlarged window to perform joint sparse representation, while a larger *m* increases the completeness of the dictionary. It is important to find a suitable dictionary size. *n* and *m* values which are too small cause large reconstruction errors and loss of details, while overly large *n* and *m* easily cause artifacts and take more time.

In this experiment, the decomposition level *K* was set to 5 while other parameters were set to default values. All the dictionaries used in this experiment were trained with 100,000 natural image patches for 180 iterations by the K-SVD algorithm, as suggested in [34]. All 16 sets of source images were tested. The average values of the objective metrics and the average time cost were compared. First, *m* was fixed to 512 and *n* was set to $\left\{36,64,100\right\}$, respectively. Then, *n* was fixed to 64 and *m* was set to $\left\{128,256,512\right\}$, respectively. The results are shown in Table 1 and Table 2.

It can be seen that the size of 64×512 achieved the highest MI, $Q^{AB/F}$ and NCIE in both experiments, and its FMI values were both the second highest. At the same time, its time cost was not too high compared to others. Therefore, it is reasonable to choose the size of the JSR dictionary as 64×512.

#### 4.2.2. Number of Decomposition Level

This experiment tested the effect of different decomposition levels *K* on the fusion performance. For the innovation image of each source image, RGF decomposition generates K−1 detail layers and one base layer. The base layer can be considered as the last detail layer with the highest degree of blur. At the same time, K−1 weight maps are generated by JBF to guide the fusion of detail layers. To ensure that at least one detail layer exists, the minimum value of *K* should be 2.

**Figure 8:**
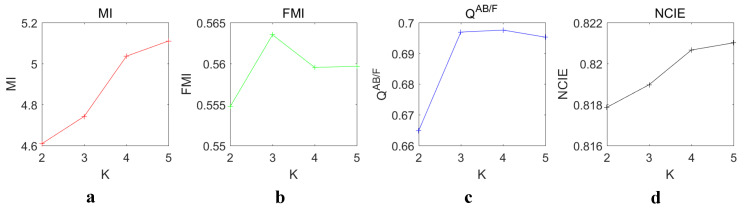
Objective evaluation of different decomposition level *K*. (**a**–**d**) The values of MI, FMI, $Q^{AB/F}$ and NCIE, respectively.

In this experiment, *K* was set to $\left\{2,3,4,5\right\}$, respectively. The other parameters were set to default. The 64×512 dictionary was used for JSR as mentioned before. All 16 sets of source images were tested. The average values of the objective metrics were compared. The results are shown in Figure 8.

As *K* increases, MI and NCIE also increase. The highest values of MI and NCIE were at K=5, but both the growth rates from K=4 to K=5 were low. Both the highest growth rates were from K=3 to K=4. FMI reached the highest value at K=2. After reaching the highest value, the value dropped. NCIE reached the highest value at K=3. Except from K=2 to K=3, the growth rate of NCIE was approximately equal to zero. Since two metrics reached the maximum value at K=5, and the growth rates of all four metrics tended to be zero at K=5, it is reasonable to choose K=5 as the default decomposition level.

We also compared the calculation times required for different decomposition levels. The comparison results are shown in Table 3.

As K increases, the time cost increases at a very slow rate. Compared with the time cost of JSR decomposition, the time cost of MSD using RGF is very small. The 5-level decomposition is only about 6% slower than the 2-level decomposition. It is worthwhile to trade these time costs for performance improvements. Overall, it is reasonable to choose K=5 as the default decomposition level.

### 4.3. Validity of Our Combination Strategy

**Figure 9:**
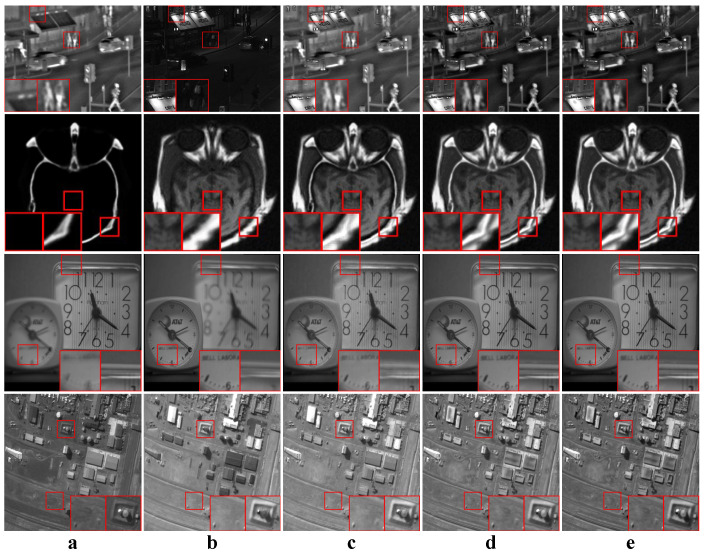
Some fused images of JSR, RGF and our proposed method. (**a**,**b**) Source images; (**c**) The fused results of JSR; (**d**) The fused results of RGF; (**e**) The fused results of our proposed method.

In this experiment, our proposed method was compared to original JSR-based [26] and RGF-based [18] image fusion methods to validate the effectiveness of our combination strategy. Our method adopted the aforementioned default parameters, and the JSR-based and RGF-based methods adopted the default parameters from their papers. All four categories of source images were tested. The average values of the objective metrics of each category were compared separately. Some examples of the fused images are shown in Figure 9. The average values of the objective metrics are shown in Table 4.

According to Figure 9, RGF and our method perform similarly in terms of subjective effects. But the subjective effect of JSR is not as good as the other two. For fused images of JSR, the details in some images are smoothed, and some images have local artifacts. The objective metrics in Table 4 show that for images other than medical ones, our method is the best. This means that the fused images of our method have higher information entropy and visual quality. In the results of medical images, our method achieves best MI and $Q^{AB/F}$, while JSR achieves best FMI and NCIE. However, according to the medical image fusion example in Figure 9, our method generates clearer details than the JSR method.

Our method combines JSR and RGF for better image fusion performance, while JBF can be regarded as a special form of RGF. First, JSR decomposition is used to obtain two innovation images and a common image. The two innovation images contain the complementary information of the two source images, which have more information entropy and less redundancy. The complementary information is what really needs to be fused. Since the common image contains the redundant information shared by the two source images, it should be directly included in the fused image without modification. Second, the combination of JBF and RGF for image fusion was proven to be very effective. RGF is used for multi-scale edge-preserving decomposition, which makes the most use of details and textures. JBF is used to obtain the corresponding weight maps, which take spatial consistency well into account and reduces local artifacts [18]. To further improve the quality of fusion, the innovation images generated by JSR were used as the input images of RGF and the guidance images of JBF. The MSD using RGF can extract complementary details of the innovation images at different levels. Meanwhile, using the innovation images as the guidance images of JBF can make the weight maps balance the contribution of the innovation images well. Then, different strategies are used to fuse the detail layers and the base layer to make the fused innovation image have high contrast and more details. Finally, adding the common image directly to the fused innovation image ensures that common information can be preserved.

Overall, our method is superior to the single JSR-based and RGF-based methods. This experiment proves the validity of our proposed combination strategy.

### 4.4. Comparison with Other Methods

In order to prove the superiority of the proposed method, we compare it with 13 other image fusion methods here. The comparative methods include the adaptive sparse representation (ASR) [35], the convolutional sparse representation (CSR) [36], curvelet transform (CVT) [37], dual-tree complex wavelet transform (DTCWT) [8], the gradient transfer fusion (GTF) [38], the hybrid multi-scale decomposition (H-MSD) [39], the convolutional neural network (CNN) [40], Laplacian pyramid (LP) [5], the general framework based on multi-scale transform and sparse representation (MSSR) [34], the multi-resolution singular value decomposition (MSVD) [41], nonsubsampled contourlet transform (NSCT) [9], the visual saliency map and weighted least square optimization (WLS) [42] and the fast filtering image fusion (FFIF) [43]. All these methods were given default parameters from in their related papers. For our proposed method, the dictionary size was 64×512, the decomposition level *K* was set to 5 and the other parameters were set to defaults.

**Figure 10:**
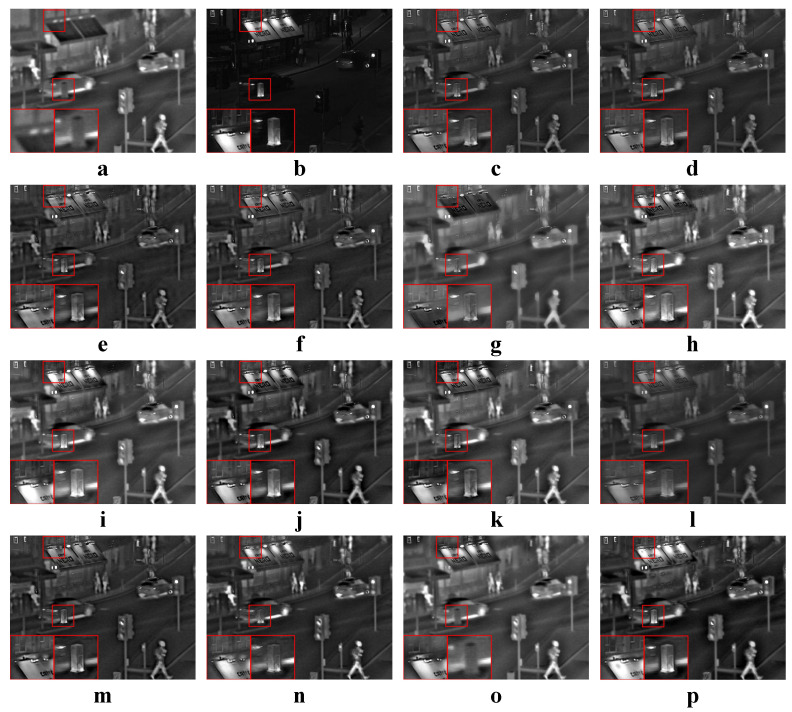
Examples of the fusion results of infrared-visible images. (**a**,**b**) Source images; (**c**–**p**) The fused results of ASR, CSR, CVT, DTCWT, GTF, H-MSD, CNN, LP, MSSR, MSVD, NSCT, WLS, FFIF, and our proposed method, respectively.

**Figure 11:**
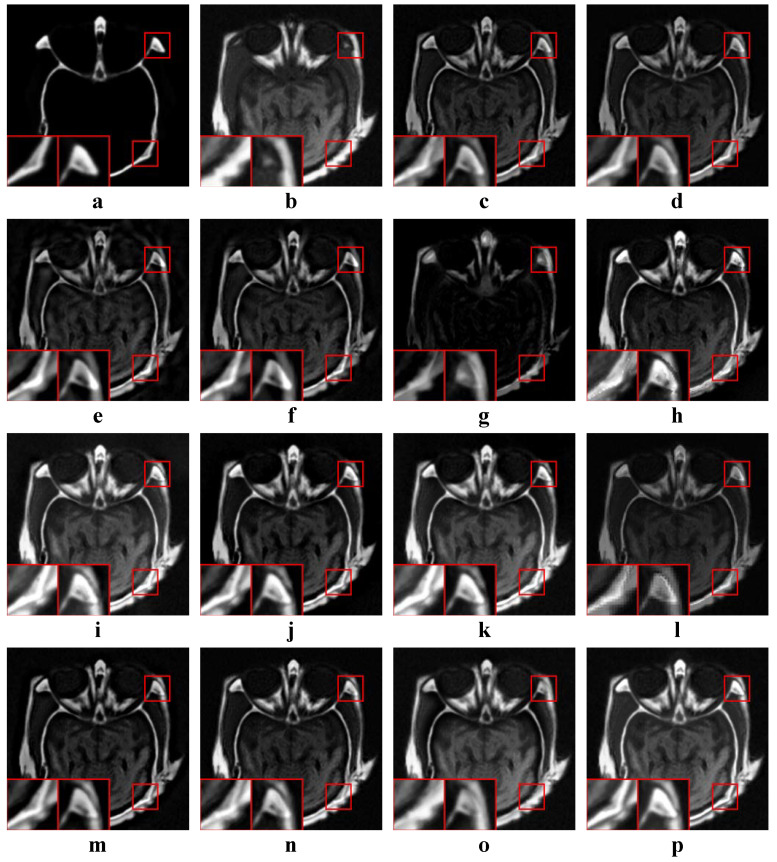
Examples of the fusion results of medical images. (**a**,**b**) Source images; (**c**–**p**) The fused results of ASR, CSR, CVT, DTCWT, GTF, H-MSD, CNN, LP, MSSR, MSVD, NSCT, WLS, FFIF, and our proposed method, respectively.

**Figure 12:**
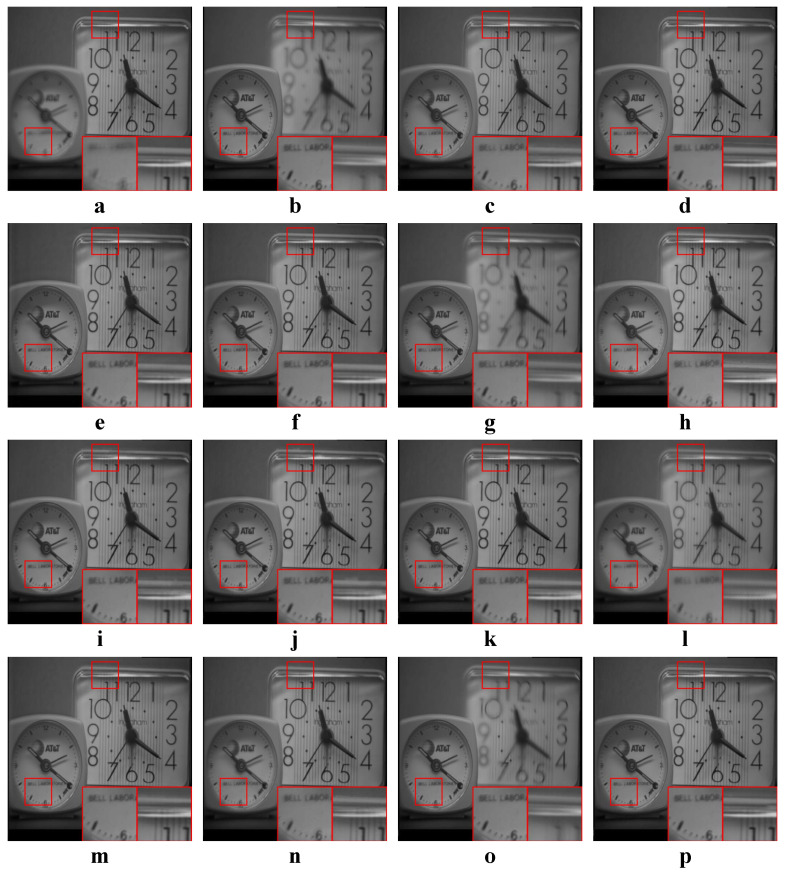
Examples of the fusion results of multi-focus images. (**a**,**b**) Source images; (**c**–**p**) The fused results of ASR, CSR, CVT, DTCWT, GTF, H-MSD, CNN, LP, MSSR, MSVD, NSCT, WLS, FFIF, and our proposed method, respectively.

**Figure 13:**
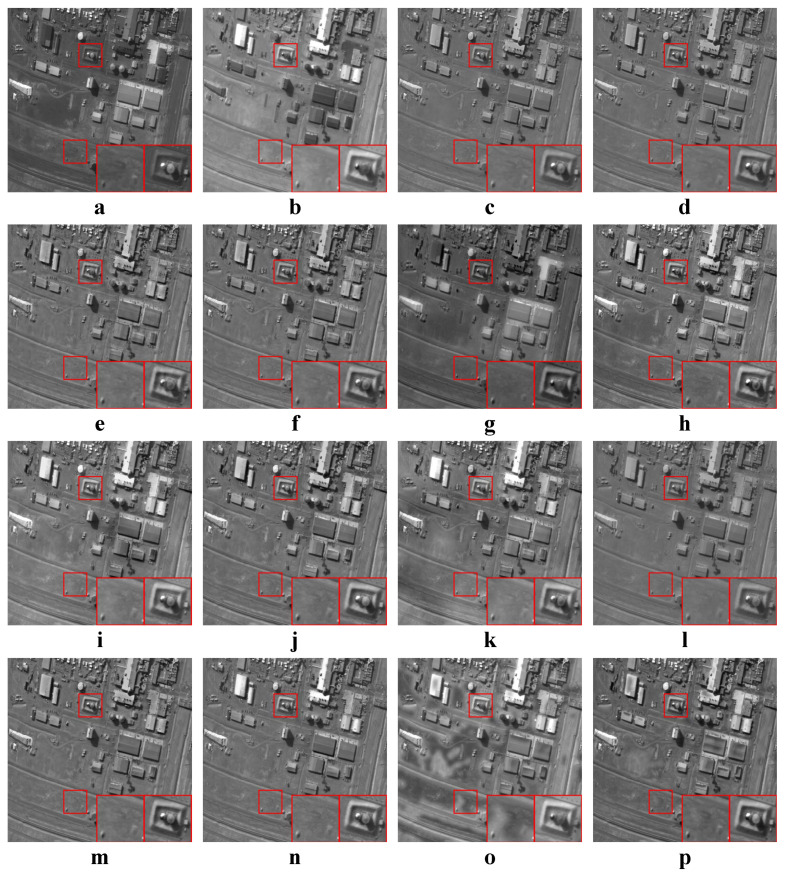
Examples of the fusion results of remote sensing images. (**a**,**b**) Source images; (**c**–**p**) The fused results of ASR, CSR, CVT, DTCWT, GTF, H-MSD, CNN, LP, MSSR, MSVD, NSCT, WLS, FFIF, and our proposed method, respectively.

In this experiment, all four categories of source images were tested. The average values of the objective metrics of each category were compared separately. Some examples of the fused images are shown in Figure 10, Figure 11, Figure 12 and Figure 13. The average values of the objective metrics are shown in Table 5, Table 6, Table 7 and Table 8.

#### 4.4.1. Analysis of Infrared–Visible Results

According to Figure 10, our method produces clear structures of the windows and the barrel. GTF produces the sharpest silhouettes and structures. However, in the GTF result, the light under the windows in the visible image is not fused, which reduces the local contrast and makes the letters unclear. CNN performs similarly to our method. The results of other methods either have unclear structures or have low contrast.

According to Table 5, our method is the best on $Q^{AB/F}$ and the second best on the other three metrics. FFIF is the best on MI, FMI and NCIE, while it is the second best on $Q^{AB/F}$. However, the visual quality of FFIF is not so good. Some information in the visible image is not fused at all. Some details from the visible image are missing in the fused image. Although the metrics show that the results of FFIF have high information entropy, this is in exchange for the decline in visual quality. In contrast, our method achieves a balance between visual quality and information entropy.

#### 4.4.2. Analysis of Medical Results

According to Figure 11, our method produces both high contrast and clear structures. With the exception of CNN, MSSR, FFIF and our method, all other methods make the texture from the middle part of (b) unclear due to low contrast. However, the structures produced by CNN, MSSR and FFIF are not as clear as our method.

According to Table 6, our method is the best on $Q^{AB/F}$ and the second best on MI and NCIE. FFIF is the best on MI, FMI and NCIE, while it is the second best on $Q^{AB/F}$. ASR is the second best on FMI. However, the edges and structure of the FFIF result are unclear. The structures of the two source images are mixed together, and it looks confusing. The result of ASR has low contrast and the middle part of the fused image is not clear. As pointed out by $Q^{AB/F}$, our method has the best visual quality. At the same time, our method also has high information entropy.

#### 4.4.3. Analysis of Multi-Focus Results

According to Figure 12, our method preserves the clear letters and edges well. Letters in ASR, H-MSD, MSVD and WLS results are not very clear. The edges of the right clock in MSSR and NSCT are not well preserved. In the CSR result, there is an obvious artifact above the clock edge. The fusion results of GTF and FFIF are very poor, the clock on the right is very fuzzy, and the information in the source image (**a**) is hardly fused. CVT, DTCWT, CNN, LP perform similarly to our method.

According to Table 7, our method is the best on $Q^{AB/F}$ and the second best on the other three metrics. FFIF is the best on MI, FMI and NCIE. CNN is the second best on $Q^{AB/F}$. However, as mentioned earlier, the fusion quality of FFIF for some multi-focus images is very poor. In this case, high values of the metrics are not so convincing. Our method can achieve high values of metrics while ensuring visual quality.

#### 4.4.4. Analysis of Remote Sensing Results

According to Figure 13, our method preserves the textures on the ground and the structures of the buildings well. ASR and MSVD fail to preserve the straight line structures in the right box. Except our method, all other methods do not preserve the textures on the ground well.

According to Table 8, our method is the best on $Q^{AB/F}$ and the second best on the other three metrics. FFIF is the best on MI, FMI and NCIE, while it is the second best on $Q^{AB/F}$. However, the results of FFIF suffer from spatial inconsistencies, and there are many discontinuous black patches on the ground. In contrast, our method has the best visual quality while having high information entropy.

#### 4.4.5. Summary of the Analysis

According to Figure 10, Figure 11, Figure 12 and Figure 13 and the $Q^{AB/F}$ metric, our method is the best in terms of visual quality compared with the other 13 methods. The MI, FMI and NCIE metrics of our method are slightly lower than those of FFIF. Although the metrics show that the fused images of FFIF have higher information entropy, FFIF does not make full use of the information in each source image. This makes the visual quality of the FFIF method not so good. In contrast, our method achieves a good balance between visual quality and information entropy.

Overall, this experiment demonstrates that our method is comparable to or better than state-of-the-art methods both in visual and objective evaluations.

## 5. Conclusions

In this paper, a JSR and RGF based image fusion method is proposed. Our method uses JSR for image decomposition, reduces the correlation and highlights the complementary information between source images. This improves the low information entropy caused by the redundant information between source images. Multi-scale decomposition using RGF can remove small structures while preserving obvious edges in the innovation images. It can extract complementary details of the innovation images at different levels. Using weight maps to balance the contribution of the innovation images can suppress the artifacts that may be brought by JSR. The innovation images are used to guide the optimization of the weight maps so that the fused image can have high contrast. The fusion of innovation images is performed according to optimized weight maps to ensure the spatial consistency of the fused innovation image. Finally, adding the common image directly to the fused innovation image without processing ensures that the common information contained in the two source images can be well retained. Experimental results demonstrate that our main contributions have been achieved, and our method can achieve better performance than state-of-the-art methods.

## Figures and Tables

**Table 1:** Objective evaluation of different *n* values for the JSR dictionary. The best and second best results of each metric are marked in red and bold, respectively.

Metric	36×512	64×512	100×512
MI	5.3075	5.3377	**5.3179**
FMI	0.5650	**0.5600**	0.5556
$Q^{AB/F}$	0.6971	0.6978	**0.6974**
NCIE	0.8224	0.8226	**0.8225**
Time	163.74	398.33	1182.71

**Table 2:** Objective evaluation of different *m* values for the JSR dictionary. The best and second best results of each metric are marked in red and bold, respectively.

Metric	64×128	64×256	64×512
MI	5.2805	**5.3309**	5.3377
FMI	0.5538	0.5638	**0.5600**
$Q^{AB/F}$	0.6936	**0.6973**	0.6978
NCIE	0.8223	**0.8225**	0.8226
Time	176.07	251.15	398.33

**Table 3:** Time cost of different decomposition levels.

K	2	3	4	5
Time	374.70	376.97	381.58	398.33

**Table 4:** Objective evaluation of JSR, RGF and our method. The best and second best results of each metric are marked in red and bold, respectively.

Category	Metric	JSR	RGF	OURS
Infrared-visible	MI	3.6597	**3.7839**	4.2105
	FMI	0.4946	**0.5456**	0.5477
	$Q^{AB/F}$	0.6130	**0.6644**	0.6652
	NCIE	0.8106	**0.8113**	0.8143
Medical	MI	**4.1862**	4.0194	4.2164
	FMI	0.5439	**0.5278**	0.5228
	$Q^{AB/F}$	0.6177	**0.6768**	0.6800
	NCIE	0.8133	0.8119	**0.8130**
Multi-focus	MI	6.9542	**8.8911**	8.9213
	FMI	0.5475	**0.6316**	0.6324
	$Q^{AB/F}$	0.7449	**0.7890**	0.7891
	NCIE	0.8316	**0.8465**	0.8467
Remote sensing	MI	2.9600	**3.7494**	4.0035
	FMI	0.4555	**0.5337**	0.5370
	$Q^{AB/F}$	0.5846	**0.6508**	0.6567
	NCIE	0.8082	**0.8145**	0.8165

**Table 5:** Objective evaluation of infrared-visible image fusion. The best and second best results of each metric are marked in red and bold, respectively.

**Metric**	**ASR**	**CSR**	**CVT**	**DTCWT**	**GTF**	**H-MSD**	**CNN**
MI	2.7134	2.7878	2.2697	2.3902	2.5465	2.6970	2.9490
FMI	0.5202	0.4510	0.4596	0.4892	0.4874	0.4324	0.4818
$Q^{AB/F}$	0.5986	0.5890	0.5512	0.5796	0.4994	0.5686	0.6290
NCIE	0.8064	0.8066	0.8052	0.8055	0.8061	0.8064	0.8072
**Metric**	**LP**	**MSSR**	**MSVD**	**NSCT**	**WLS**	**FFIF**	**OURS**
MI	2.6575	3.4726	2.9739	2.4802	2.7887	4.9717	**4.2105**
FMI	0.5003	0.5044	0.3972	0.4988	0.4339	0.5775	**0.5477**
$Q^{AB/F}$	0.6366	0.6065	0.4123	0.6144	0.5574	**0.6405**	0.6652
NCIE	0.8062	0.8107	0.8072	0.8057	0.8065	0.8226	**0.8143**

**Table 6:** Objective evaluation of medical image fusion. The best and second best results of each metric are marked in red and bold, respectively.

**Metric**	**ASR**	**CSR**	**CVT**	**DTCWT**	**GTF**	**H-MSD**	**CNN**
MI	3.4473	3.3705	2.6794	2.9084	2.9051	3.2624	3.5522
FMI	**0.5638**	0.5087	0.3534	0.4478	0.5125	0.4738	0.5152
$Q^{AB/F}$	0.6037	0.5976	0.5170	0.5488	0.4288	0.5639	0.6416
NCIE	0.8092	0.8090	0.8069	0.8075	0.8076	0.8086	0.8097
**Metric**	**LP**	**MSSR**	**MSVD**	**NSCT**	**WLS**	**FFIF**	**OURS**
MI	3.2668	3.6737	3.5279	3.1658	3.5519	4.6729	**4.2164**
FMI	0.5243	0.5406	0.4731	0.5063	0.4907	0.6086	0.5228
$Q^{AB/F}$	0.6384	0.6422	0.4713	0.6220	0.5914	**0.6535**	0.6800
NCIE	0.8085	0.8102	0.8097	0.8082	0.8097	0.8151	**0.8130**

**Table 7:** Objective evaluation of multi-focus image fusion. The best and second best results of each metric are marked in red and bold, respectively.

**Metric**	**ASR**	**CSR**	**CVT**	**DTCWT**	**GTF**	**H-MSD**	**CNN**
MI	7.5714	7.6874	7.2197	7.4468	7.6793	7.5452	8.5647
FMI	0.6022	0.4586	0.5643	0.5942	0.5963	0.5534	0.6065
$Q^{AB/F}$	0.7746	0.7570	0.7571	0.7710	0.6210	0.7477	**0.7835**
NCIE	0.8367	0.8363	0.8343	0.8360	0.8397	0.8363	0.8441
**Metric**	**LP**	**MSSR**	**MSVD**	**NSCT**	**WLS**	**FFIF**	**OURS**
MI	7.9235	7.8695	6.5958	7.5796	7.3741	9.2663	**8.9213**
FMI	0.6057	0.5987	0.4236	0.5963	0.5680	0.6558	**0.6324**
$Q^{AB/F}$	0.7829	0.7807	0.6212	0.7795	0.7647	0.7403	0.7891
NCIE	0.8390	0.8385	0.8299	0.8367	0.8349	0.8533	**0.8467**

**Table 8:** Objective evaluation of remote sensing image fusion. The best and second best results of each metric are marked in red and bold, respectively.

**Metric**	**ASR**	**CSR**	**CVT**	**DTCWT**	**GTF**	**H-MSD**	**CNN**
MI	2.0875	2.2285	1.8935	1.9541	1.7620	2.1677	3.6505
FMI	0.5057	0.4519	0.4305	0.4590	0.4967	0.4141	0.4741
$Q^{AB/F}$	0.5375	0.5908	0.5526	0.5802	0.4661	0.5428	0.6194
NCIE	0.8047	0.8052	0.8044	0.8045	0.8037	0.8055	0.8143
**Metric**	**LP**	**MSSR**	**MSVD**	**NSCT**	**WLS**	**FFIF**	**OURS**
MI	2.1795	3.1134	2.0510	2.0314	2.1937	4.5241	**4.0035**
FMI	0.4784	0.4646	0.3010	0.4757	0.4199	0.5473	**0.5370**
$Q^{AB/F}$	0.6173	0.5941	0.4055	0.6119	0.5389	**0.6298**	0.6567
NCIE	0.8051	0.8110	0.8044	0.8046	0.8050	0.8209	**0.8165**
